# Understanding plant pathogen interactions using spatial and single-cell technologies

**DOI:** 10.1038/s42003-023-05156-8

**Published:** 2023-08-04

**Authors:** Jie Zhu, Alba Moreno-Pérez, Gitta Coaker

**Affiliations:** Department of Plant Pathology, University of California, Davis, One Shields Avenue, Davis, CA 95616 USA

**Keywords:** Effectors in plant pathology, Plant genetics

## Abstract

Plants are in contact with diverse pathogens and microorganisms. Intense investigation over the last 30 years has resulted in the identification of multiple immune receptors in model and crop species as well as signaling overlap in surface-localized and intracellular immune receptors. However, scientists still have a limited understanding of how plants respond to diverse pathogens with spatial and cellular resolution. Recent advancements in single-cell, single-nucleus and spatial technologies can now be applied to plant*–*pathogen interactions. Here, we outline the current state of these technologies and highlight outstanding biological questions that can be addressed in the future.

## Abstract (web summary)

This review highlights the importance of comprehending plant-pathogen interactions at the single-cell and spatial levels, while also providing an overview of relevant cutting-edge technologies.

## Introduction

Plants can be infected by diverse pathogenic organisms that modulate host cells to enable their growth and dissemination. Pathogens deploy a variety of manipulation strategies that can vary during the course of infection. These strategies include plant defense suppression as well as deployment of toxins and degradative enzymes to facilitate colonization and nutrient release^1^. Some pathogens can directly penetrate and invade plant tissues, while others gain entry through wounds or natural openings. Vector-borne pathogens can be delivered directly into vascular tissues by different groups of piercing–sucking insects. Each mechanism of tissue invasion brings the pathogen into contact with different cell and tissue types^1–3^.

Distinct pathogen infection stages are simultaneously observed in a leaf^4^. Pathogen distribution in plants is not homogeneous, generating unequal symptom development^5–7^. Most previous studies investigating plant*–*pathogen interactions were carried out using whole plants or complex tissue types. There are significant differences between single-cell and whole-tissue responses, suggesting that the response observed at the tissue level is an average of oscillations occurring between pathogen-targeted and untargeted cells^8^. Recent technological advancements enable scientists to investigate plant and pathogen responses at single-cell resolution or in a spatial context^9–11^. These advancements will facilitate a more holistic understanding of cellular responses and variability within a tissue.

Plants possess an innate immune system, which consists of surface localized pattern recognition receptors (PRRs) and intracellular nucleotide-binding leucine-rich repeat receptors (NLRs)^12^. PRRs can recognize conserved microbe- and damage-associated molecular patterns (MAMPs and DAMPs, respectively), resulting in PRR-triggered immunity (PTI). Plant NLR immune receptors recognize secreted pathogen effectors inside cells, inducing effector-triggered immunity (ETI)^12^. Although PRRs and NLRs are structurally distinct and can recognize different pathogen components, they share significant overlap in downstream signaling, such as mitogen-activated protein kinase (MAPK) cascades, calcium flux, a burst of reactive oxygen species (ROS), transcriptional reprogramming, and phytohormone signaling^13^. Recently, it was shown that PTI and ETI mutually potentiate each other to mediate robust resistance^14,15^. Advancements in single-cell and spatial technologies have the potential to elucidate cellular responses in pathogen-targeted and adjacent cells.

Although immune signaling has been extensively studied, scientist still lack an understanding of the spatial expression of immune receptors across diverse plant tissues. For many years, we assumed that all plant cells were immune competent. Recently, it has been demonstrated that only a restricted subset of *Arabidopsis* root zones directly respond to the flagellin MAMP in the absence of damage^16,17^. Detailed characterization of response competent plant cells during the infection process is necessary to understand the mechanisms that regulate disease progression.

Here, we discuss the challenges and potential of studying pathogen–plant interactions from a spatial and single-cell point of view. First, we briefly introduce the current state of spatial and single-cell technologies. Next, we immerse ourselves in how these technologies can be used to address outstanding biological questions.

### Technologies for profiling responses using single-cell, single-nucleus and spatial transcriptomics

Studies at the level of tissues or organs have advanced our understanding of pathogen perception and plant signaling in compatible (susceptible) and incompatible (immune or non-host) interactions^12,18,19^. However, many important questions still remain. How do different plant cell types respond to pathogen infection? How do pathogen-targeted cells communicate with their neighbors? What transcripts, proteins and metabolites are masked in bulk analyses but play essential roles during plant*–*pathogen interactions? Recent advances in single-cell/-nucleus and spatial analyses facilitate the investigation of these foundational questions^9–11,20–23^.

Single-cell RNA sequencing (scRNA-seq) enables gene expression profiling in individual cells^22,24–26^. scRNA-seq was first developed to analyze the cellular transcriptome from a single mouse blastomere^27^. In 2012, this technology was used to profile hundreds of cells; in 2015, transcriptional profiling of 44,808 mouse cells was achieved^28,29^. In 2016, scRNA-seq was adopted to plant root tissue^30^. Since then, this technology has been used to understand plant development and acclimatization to changing environments^22,31,32^.

**Fig. 1:**
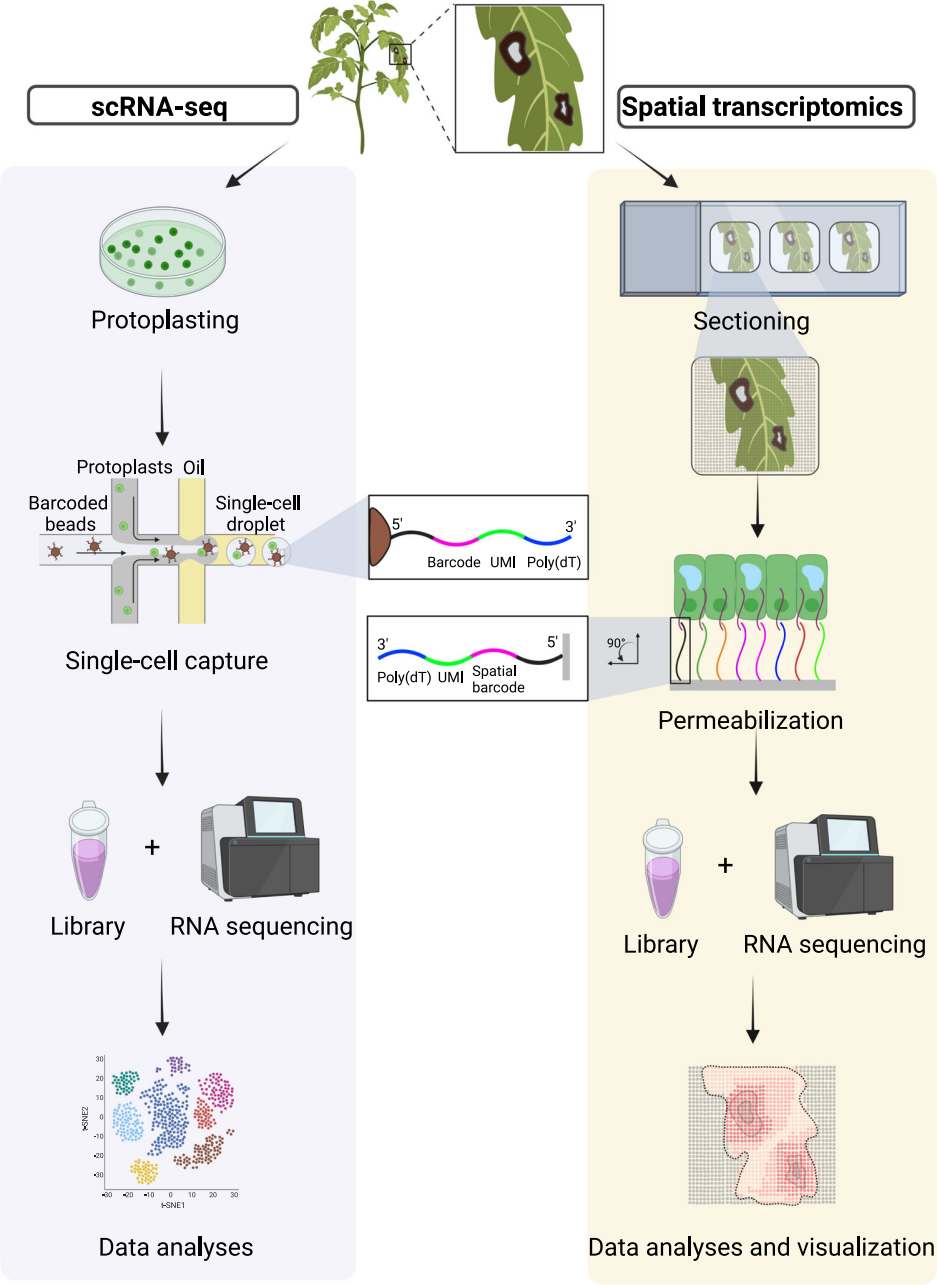
A schematic workflow of single-cell RNA sequencing and spatial transcriptomics. For scRNA-seq, individual cells are isolated through enzymatic digestion from intact tissue to generate protoplasts. Microfluidics are used to separate and encapsulate single protoplasts in droplets with barcoded beads. Each barcoded bead is coated with DNA probes, which contain poly (dT) to capture mRNAs, a unique molecular identifier (UMI) and a cell-specific barcode. Cell lysis then occurs, and mRNAs are hybridized to probes and reverse transcribed on beads within droplets. Finally, a library of barcoded cDNA from thousands of single cells is sequenced to generate single-cell transcriptomes. For spatial transcriptomics, freshly frozen plant tissue is sectioned onto a spatial transcriptomics slide. Capture zones on the slide contain thousands of spots. Each spot consists of probes containing a unique spatial barcode, unique molecular identifier (UMI), and poly (dT) region to capture transcripts. Transcripts are released through permeabilization, hybridized to probes and reverse transcribed to incorporate spatial barcodes. The generated cDNAs carrying spatial information are cleaved from the slide and used to prepare a library for sequencing. The sequenced reads are spatially mapped based on spatial barcodes. Created with BioRender.com.

The most widely used scRNA-seq technology is droplet-based, which combines microfluidics with barcodes to allow parallel and high-throughput transcriptome profiling of individual cells (inDrop, Drop-seq, and 10× Genomics; Fig. 1)^22,25,29^. A plant cell consists of transcripts in both the cytoplasm and nucleus. The half-life of a transcript can be up to 24h^33^. Therefore, the cellular transcriptome can capture gene expression over time. RNA-seq can be performed with nuclei (snRNA-seq) or isolated individual cells (scRNA-seq). In *Arabidopsis*, 14% of total genes exhibited differential expression between these two methods^34^. snRNA-seq detected fewer transcripts than scRNA-seq, but it still profiled ~90% of the total transcripts detected in scRNA-seq. One advantage of snRNA-seq is this approach enables profiling cells that are difficult to enzymatically digest. Overall, both snRNA-seq and scRNA-seq exhibit high correlation with bulk RNA-seq (*r* = 0.7–0.8) and reflect the expression pattern in intact tissue^34^. For snRNA-seq, nuclei can be isolated using a variety of approaches on fresh or frozen tissue^35–37^. For scRNA-seq, individual cells are isolated through enzymatic digestion from plant tissues (Fig. 1)^22^. The protoplasting process results in transcriptional changes, which can also affect transcripts in a cell-type/state-specific manner^38,39^. Thus, it is essential to include bulk RNA-seq of the entire tissue as a control to exclude genes affected by the protoplasting process. Next, isolated cells are separated and encapsulated in droplets with barcoded beads using microfluidics. Subsequently, cell lysis and cDNA barcoding occur within droplets. Finally, transcripts are sequenced in parallel on the Illumina platform and demultiplexed (Fig. 1)^22,26^.

In addition to droplet-based methods, plate-based methods are typically combined with fluorescence activated cell sorting (FACS) to sort cells of interest into individual wells in a plate^22,24,25^. FACS is a well-established method used in conjunction with fluorescent reporter lines to capture specific groups of cells. For example, FACS has been used to sort specific phloem and root cell types that were then analyzed by scRNA-seq^40^. While plate-based methods have lower numbers of profiled cells, they have the highest sensitivity to study rare transcripts or limited tissues^22,24^. Laser-capture microdissection is another method to capture tens or hundreds of cells under direct microscopic visualization^41^. Recent advancements in combinatorial barcoding strategies (SPLiT-seq and sci-RNA-seq) enable transcriptomic profiling from an exponentially scalable number of individual cells or nuclei^42–44^.

Tissue dissociation is required for single-cell isolation, but results in damage and a loss of spatial content within a tissue, which is critical to understand cellular function^20^. Spatial transcriptomics (ST) was developed to preserve spatial information while profiling cellular gene expression at high resolution. ST includes spot-based and fluorescence in situ hybridization (FISH)-based methods. Spot-based ST utilizes spot-specific barcodes anchored in an array to capture and label mRNAs from tissue cryosections (Fig. 1)^21,45^. This has been commercialized and applied in human/animal tissues at single-cell resolution^46^. Recently, spot-based ST has been extended to plant tissues, including *Populus tremula* leaf buds, *Arabidopsis* and *Picea abies* cones^9,21,37,47^. For the Visium spatial transcriptomics technology (10× Genomics), the center-to-center distance between neighboring spots is 100 µm which is bigger than the average size of a single plant cell (10–100 µm)^21,45^. The larger distance between spots will decrease resolution for a single tissue piece, but could ultimately be compensated by including additional capture areas. Another spot-based ST technology, Stereo-seq, has been applied to *Arabidopsis* leaves. Stereo-seq docks a nanoball as a spot, which significantly reduces the distance between neighboring spots to 500 nm^47^. In addition to spot-based methods, the FISH-based ST have been used in plant research^11,37^. For example, plant hybridization-based targeted observation of gene expression map (PHYTOMap), was developed to spatially resolve expression of dozens of genes at a single-cell level in whole-mount *Arabidopsis* root tissue^11^. PHYTOMap is more affordable than commercially available spatial transcriptomic platforms, such as Molecular Cartography (Resolve Biosciences) and MERSCOPE (Vizgen).

Transcriptomic approaches allow researchers to analyze cellular changes indirectly, but proteomic and metabolic profiling provides a more direct characterization of a cell’s functional output^48^. Advancements in single-cell and spatial technologies for characterizing proteomic and metabolic states are not as advanced as transcriptomic analyses due to technical challenges^48–50^. Technical challenges imposed by the nature of metabolites and proteins include: different chemistries required for different analyte types, diverse proteins and metabolites with varied abundance and the ability of metabolites to leak out of cells. Furthermore, the detection limit for protein and metabolite quantification is often higher than for transcriptional profiling and metabolite identification depends on the presence of a library of known compounds^51^. Recently, spatially resolved proteome analyses using laser capture microdissection coupled with nanodroplet-based sampling was used to profile proteins from ~8–15 parenchyma cells of the tomato fruit pericarp, but was limited to ~400 proteins^52,53^. Spatial metabolomics has enabled profiling of a single soybean root nodule inoculated with *Bradyrhizobium japonicum*^10^. Mass spectrometry imaging can resolve spatial distribution and quantification of targeted plant metabolites at as high as single-cell resolution^54,55^.

### Investigating the response of different plant tissues during pathogen infection

Plant pathogens employ a wide variety of invasion strategies and can colonize different tissue types. Most research has focused on leaf invasion through open stomata and wounds or by direct penetration of the leaf epidermis^1,19^. However, several devastating bacterial, fungal, and viral pathogens can invade vascular tissue^2,3,56^. For example, soilborne pathogens are frequently xylem colonizing and must navigate multiple cell types in the root to reach the vascular system^57^. Scientists still have a poor understanding of the specific cell types that are manipulated by pathogens, and how these cells can respond to pathogen infection.

While roots are immune signaling competent, different zones within the root respond differentially to MAMPs or pathogen infection^58–62^. Using fluorescent reporter lines for immune marker genes and a PRR receptor, differentiated outer cell layers in the *Arabidopsis* root display dampened MAMP sensitivity until they encounter damage, which locally boosts immune responsiveness^16,17^. These spatially restricted root responses may be due to the prevalence of microbial communities in the rhizosphere^17^. RNA-seq analyses also revealed NLR gene expression varies between organs in a species-specific manner^63^. A recent study generated a transcriptome atlas of *Arabidopsis* from seed-to-seed that encompasses all major organs using snRNA-seq^37^. This important resource can be used to query expression of PRRs, NLRs and immune signaling networks to gain greater insight into developmental and tissue specific regulation of immune competence.

The ability to profile the response to pathogen infection with spatial and cellular resolution will result in a more holistic understanding of how plants respond to diverse pathogens. What cell types are immune capable? How does cellular signaling change depending on cell and tissue type? Immune responses cannot be completely understood without considering the role of different cell types, plant age and developmental stage. Scientists have begun to reveal varying immune status in different cell types using a combination of sc/snRNA-seq coupled with FISH-based spatial mapping of transcripts^64,65^. For example, a significant portion of Toll/Interleukin-1 receptor NLR (TNL) genes exhibited enriched expression in the vasculature, while this was not observed for coiled-coiled NLR (CNL) and CC_R_ domain-NLRs in *Arabidopsis* upon inoculation with the fungal pathogen *Colletotrichum higginsianum*^64^. Distinct patterns of immune gene expression in particular cell populations were also detected in *Arabidopsis* plants infected with virulent and avirulent *P. syringae* using a combination of snRNA-seq, snATAC-seq, and spatial transcriptome (MERFISH) analyses^65^. For example, genes involved in systemic acquired resistance (SAR) including *ALD1*, *FMO1* and *ILL6* also showed enhanced expression in Phloem Companion Cells, indicating that this cell type may play a unique role in regulating SAR and contribute to sending systemic immune signals^65^. By unraveling cell type-specific immune responses, these studies contribute to a better understanding of the complexity and coordination of plant immune responses.

**Fig. 2:**
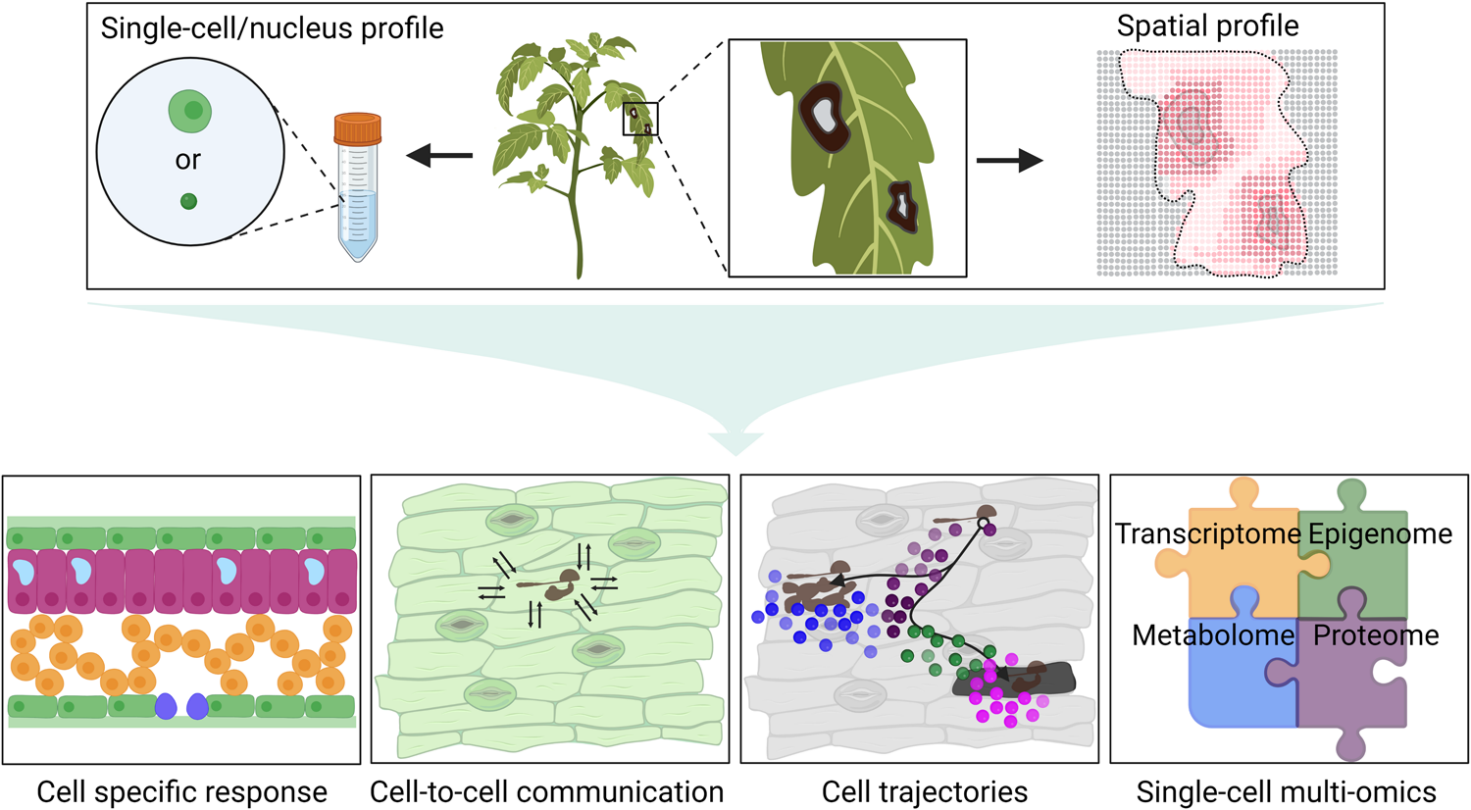
Application of cellular and spatial profiling technologies in plant-microbe interactions. A schematic diagram showing representative research areas that can be investigated by single-cell, single-nucleus and spatial profiling. A combination of different approaches will lead to a more comprehensive understanding of plant-microbe interactions. Created with BioRender.com.

Genetically encoded fluorescent reporter lines expressing immune markers in defined cell types have also been used to spatially investigate plant responses^16,17^. A combination of FACS and SMART-seq single-cell technologies enabled transcriptional profiling of protophloem sieve elements, metaphloem sieve elements, companion cells and phloem pole pericycle cells^40^. A similar approach could facilitate investigation of specific responses from rare cell types or states during pathogen infection through sc/snRNA-seq or ST (Fig. 2).

### The importance of heterogeneity within a tissue during pathogen infection

Microbial distribution is variable in and on plant tissue^66,67^. Moreover, multiple infection stages simultaneously exist within a tissue even under controlled conditions. For example, three distinct infection phases can be simultaneously observed in a single rice leaf sheath after inoculation with the fungal pathogen *Magnaporthe oryzae* (early and late biotrophic, transient necrotrophic phase)^4^. Spores of the fungal pathogen *Zymoseptoria tritici* germinate and penetrate wheat leaves for 10 days, leading to multiple asynchronized infections that exist simultaneously^7,68,69^. The plant response to asynchronous infection and uneven effector targeting will result in heterogeneous cellular responses that cannot be revealed using bulk RNA-seq analyses.

Not only is pathogen distribution variable within a tissue, but pathogens also exhibit cell-to-cell differences in gene expression during infection^70–72^. For example, the necrotrophic bacterial pathogen *Dickeya dadantii* exhibits heterogeneity in cell length and gene expression to balance virulence and vegetative growth during infection^70^. In *Pseudomonas syringae*, expression of the genes encoding the type three secretion system shows a reversible bi-stable pattern of expression that affects bacterial virulence^72^. The impact of transcriptional heterogeneity in isogenic bacterial populations cannot be studied using bulk RNA-seq analyses. Technical challenges have hindered adapting scRNA-seq technology to microbes; prokaryotic mRNAs are lower in abundance and are not polyadenylated. Nevertheless, several tools have been developed recently for single-cell bacterial RNA-seq in vitro, including microbial split-pool ligation transcriptomics (microSPLiT), probe-based bacterial sequencing (ProBac-seq), and prokaryotic expression profiling by tagging RNA in situ and sequencing (PETRI-seq)^73–75^. The combination of these tools coupled with efficient bacterial isolation methods from plant tissue, such as a density gradient centrifugation in the presence of an RNA protecting reagent^76^, could help elucidate heterogeneous microbial responses during infection.

Understanding the association between microbial distribution and the spatiotemporal regulation of plant responses during colonization remains an area that requires further exploration^77^. The spatial distribution of complex bacterial communities in plant tissue was recently reported using a novel technique called sequential error-robust FISH (SEER-FISH)^78^. Cao et al.^78^ investigated the microbial colonization patterns along the root and observed changes in the spatial relationships among different microbial taxa in synthetic communities. In natural environments, plants interact with diverse microbial communities that exhibit a wide range of behaviors, from pathogenic to mutualistic^79^. To apply the SEER-FISH method to natural communities, it is necessary to first identify the species comprising the bacterial community. Recent simultaneous detection of microbial location and plant transcripts using spatial transcriptomics highlights the heterogenicity of both microbial distribution and plant responses within the same leaf^9,65^. Future advancements in ST should enable profiling of both plant and pathogen transcriptomes, including the profiling of pathogen-targeted and adjacent cells (Fig. 2). By examining the transcriptomic changes occurring in both the plant and microbial cells during colonization, researchers can gain a comprehensive understanding of the molecular interactions and signaling processes involved in the establishment of plant–microbe associations.

Several recent studies have highlighted the importance of spatiotemporal plant responses using ST, scRNA-seq alone, or a combination of scRNA-seq and genetically encoded reporter lines^36,64,65,80–87^. Different cell types in *Medicago truncatula* roots exhibited differentially regulated gene expression in response to the bacterial symbiont *Ensifer (Sinorhizobium) meliloti*^36^. Single-cell metabolomics has also revealed metabolic heterogeneity in root nodules colonized by the symbiotic bacterium *Bradyrhizobium japonicum*^10^. Cellular heterogeneity in plant-microbe interactions can be visualized in a temporal manner using cell trajectory analyses^80–83,87^ (Fig. 2). For example, a continuum of disease progression from an immune to susceptible state within a leaf was revealed from single-cell transcriptomic profiling of a compatible interaction between *Arabidopsis* and *P. syringae*^82^. In addition, the cellular heterogeneity in plant immune response is also spatially regulated. Spatially restricted immune gene expression is correlated with pathogen distribution, with plant cells proximal to pathogen colonization exhibiting stronger immune gene expression^9,64,65,82,88^. *P. syringae* enters plant apoplast through natural openings such as stomata and accumulates in substomatal cavities. Zhu et al.^82^ found that the expression of the immune marker *FRK1* was highly induced in cells surrounding these cavities colonized by *P. syringae*. Furthermore, in *Arabidopsis*, Liu et al.^88^ observed two successive peaks of transcriptomic responses during ETI induced by the AvrRpt2 effector. These peaks represented different cellular populations with distinct gene expression patterns. The first peak corresponded to cells responding cell-autonomously to AvrRpt2, indicating a localized immune response. The second peak consisted of cells surrounding the initially responding population, suggesting a coordinated response in the neighboring cells. Future advances using a combination of single-cell and high-resolution spatial approaches will provide greater insights into cellular heterogeneity and spatiotemporal dynamics occurring during plant–microbe interactions.

Hallmarks of plant immune responses (ROS, Ca^2+^, DAMP production) act as important signaling molecules for cell-to-cell communication^89^. Global reprogramming of plant tissues for defense compromises plant growth^90^. During natural infection, plants must be able to restrict pathogen infection without completely reprogramming entire tissues. Plants can compartmentalize defense responses by spatially restricting immune responses within a leaf to a few cell layers through a local acquired resistance (LAR)^91,92^. Both transcriptional reporter lines and spatial transcriptomics have revealed spatially restricted *Arabidopsis* leaf immune responses around bacterial colonies upon ETI activation^65,82,92–95^. Furthermore, three additional immune reporter lines demonstrated expression of immune transcripts in a circular pattern of several cell layers in close proximity to *P. syringae* colonies after surface inoculation^82^. Expression of plant defense genes and microbial abundance also exhibit spatial correlations on both outdoor grown and growth-chamber inoculated *Arabidopsis* using ST^9,65^.

### Challenges and future opportunities for investigating plant-microbe interactions with spatial and cellular resolution

Despite significant advances in single-cell techniques, challenges remain when studying dynamic interactions between two or more organisms. For example, identifying, isolating and profiling plant cells invaded by pathogens remains challenging. The physical interaction between plant cells and the pathogen are removed after isolation of nuclei. Advancements in ST and strategies to tag infected cells in direct contact with the pathogen are needed. These advancements will facilitate an understanding of how pathogen-targeted plant cells communicate with their neighbors. It will also be important to profile cellular signaling in response to different pathogens at different stages of infection in different tissue types across plant genotypes to obtain a holistic understanding of the repertoire of how plants respond to pathogen infection.

Simultaneous transcriptional profiling of both the plant and pathogen remain challenging at cellular resolution. Plants, as well as pathogens, exhibit heterogeneity in their responses. Current technology captures polyadenylated eukaryotic transcripts for sequencing library preparation. This step inhibits the capture of most bacterial and viral transcripts. In order to capture thousands of cells or nuclei, most studies collect tissue from multiple pooled plants, which masks plant-to-plant variation. Other challenges include cost, data analysis, and integration^96^. This is akin to the early stages of transcriptional profiling using microarrays and RNA-seq. Developing high throughput methods with reduced cost for ST, single-cell, and snRNA-seq will facilitate broader use of these approaches.

During plant-microbe interactions, plants have evolved a finely regulated cell-to-cell communication to combat constant exposure of microbial challenges^89^. Single-cell analysis has been used to probe cell-to-cell communication in plant root cells to developmental and abiotic signals, but not yet in plant-microbe interactions^97,98^. With the development of spatial techniques, the spatially resolved transcriptomics will facilitate an understanding of cell-to-cell communication^9,65^. Moreover, incorporation of spatial coordinates in a cell trajectory may resolve differences with data generated from dissociated cells^46^.

Future investigations using spatial technologies with single-cell resolution will enable a more comprehensive understanding of how plant cells are able to communicate with their neighbors and induce robust but spatially restricted defense responses. The identification of transcriptional factors and transcripts that are masked in bulk analyses has enabled construction of gene regulatory networks (GRNs) at single-cell resolution^36,39,81^. The next frontier in plant–microbe interactions will require integration of multiple ‘omic approaches with spatial and cellular resolution, including the transcriptome, epigenome, metabolome and proteome^65,99^ (Fig. 2). Integrated analyses using single-cell multi-omics and spatial omics will provide the opportunity to holistically investigate cell-specific responses as well as reveal the landscape of plant and pathogen responsiveness with spatial resolution^22,65^. The hypotheses generated from such analyses can then be experimentally tested in the context of different plant–pathogen interactions. Ultimately, a high-resolution understanding of how plants respond to diverse pathogens will identify novel regulatory mechanisms that can be harnessed for more effective disease control.
